# Supplementary material for: Piston-Type Optical Modulator for Dynamic Thermal Radiation Tuning Applications
Source: Materials (Basel). 2021 Aug 4;14(16):4372. doi: 10.3390/ma14164372 (PMC8401391; doi:10.3390/ma14164372)
Supplement: Supplementary file 1 [file materials-14-04372-s001.zip › materials-1281414-supplementary.pdf]

Supplementary Materials: Piston-type optical modulator for dynamic thermal radiation tuning applications

Andrew Caratenuto [1] and Yi Zheng [1,2,*]

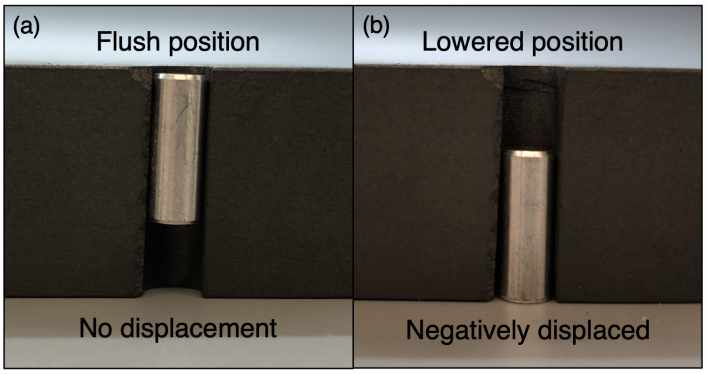

Figure S1. Example of experimental setup (cross-sectional view) illustrating two possible positions for the piston structure: (a) raised position, which promotes reflection, and (b) lowered position, which promotes absorption (cross-sectional view).

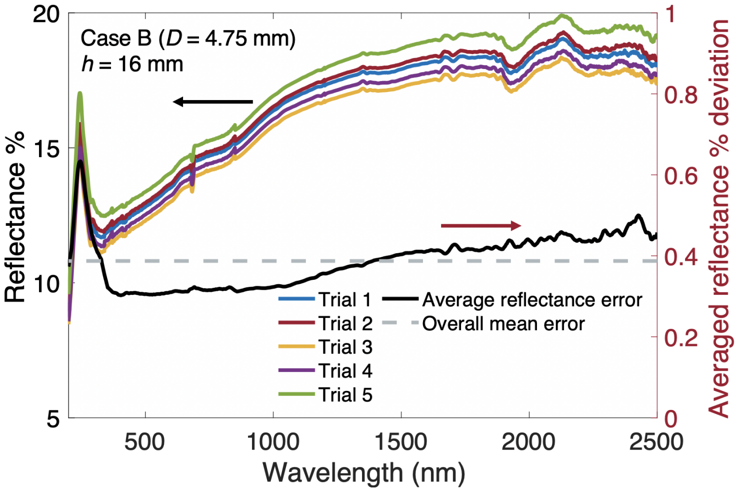

Figure S2. Five spectra of Case B at a displacement of 16 mm, tested to evaluate measurement repeatability. The figure clearly displays how only minor deviations are present based on sample placement repeatability. Each trial has a reflectance error evaluated with respect to the wavelength-dependent mean values of all five trials. The average reflectance error evaluates the mean of all these errors (25 point moving average). The overall mean error is the mean of the average reflectance error, providing an approximation for the entire wavelength range. The UV and NIR regions have slightly higher errors than the average, and the visible region has slightly lower errors than the average.

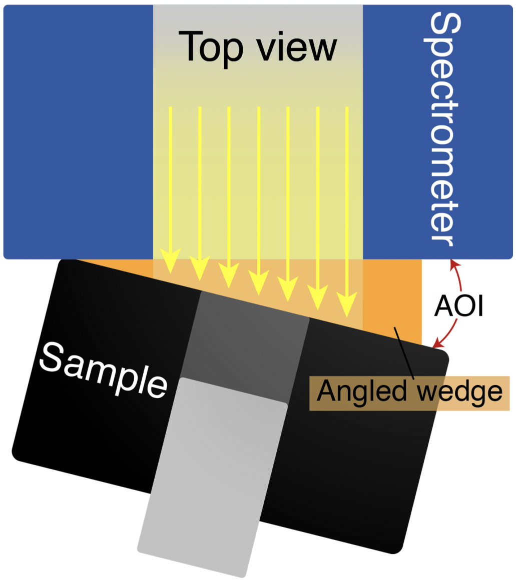

Figure S3. Schematic of experimental setup for spectra measurements at various angles of incidence (AOI). This top view illustrates how the addition of an angled wedge provides measurements at selected angles.
